# Supplementary material for: Prevalence of alcohol use by gender and HIV status in rural Uganda
Source: PLoS One. 2024 Jul 16;19(7):e0303885. doi: 10.1371/journal.pone.0303885 (PMC11251619; doi:10.1371/journal.pone.0303885)
Supplement: Attachment — Submitted filename: Prevalence of alcohol use by gender and HIV status in rural Uganda.pdf [file pone.0303885.s002.pdf]

## Reviewer report for manuscript title: Prevalence of alcohol use by gender and HIV status in rural Uganda

Dear Editor,

Thank you for granting me the opportunity to review this manuscript. Find below my suggested comments

### 1. General comments

- To me, the why of this study is not clear at all. Position this paper as a mental health related paper. It makes sense that way, otherwise, the why is not clear.

### 2. Methods

- This section is difficult to read because of how its arranged. It would be helpful to have sub headings with outcomes, exposure variables and confounding factors. It's easier to follow like that

### 3. Results

- Because there are so many components to the analysis, it would be useful to also have sub sections here. As it is, it is really difficult to go through the results section as you have to figure out on your own as a reader where the demographic characteristics ends and regression results are starting.

### 4. Discussion

- My understanding of the analysis was that the authors are trying to understand the role of gender and HIV status in alcohol consumption. But if you look at the studies that they are referencing, mostly focuses on alcohol as a risk factor for HIV infection. I was expecting the discussion to be more about the mental health related reasons that could drive those who have newly been diagnosed with HIV to get into drinking. Similarly, the cultural expectations/norms (which the authors have briefly talked about) to be more prevalent in the discussion about alcohol use and gender. Cite the studies that have looked at gender and alcohol

use.  The same thing with HIV diagnosis and alcohol consumption, cite more of such studies.

- And then the authors can also briefly look at the existing evidence which shows alcohol use as a risk factor for HIV (do not make this the focus of the discussion as it is currently, because that's not what your study focused on).

## 5. Study implication

- What are the implications of these findings? Why should policy makers care about this study at all? How does this link to the policy direction on alcohol use, mental health, and HIV care in Uganda?
